# Mechanism of Carrier Formation in P3HT-C_60_-PCBM Solar Cells

**DOI:** 10.3390/nano14171400

**Published:** 2024-08-28

**Authors:** Hiroto Tachikawa, Hiroshi Kawabata, Shigeaki Abe, Ikuya Watanabe

**Affiliations:** 1 Department of Applied Chemistry, Faculty of Engineering, Hokkaido University, Sapporo 060-8628, Japan; 2 Department of Dental and Biomedical Materials Science, Graduate School of Biomedical Sciences, Nagasaki University, Nagasaki 852-8102, Japan

**Keywords:** intermolecular interaction, excited state, carrier formation, charge transfer, charge separation

## Abstract

Solar cells convert light energy directly into electricity using semiconductor materials. The ternary system, composed of poly(3-hexylthiophene) (P3HT), fullerene (C_60_), and phenyl-C_61_-butyric-acid-methyl-ester (PCBM), expressed as P3HT-C_60_-PCBM, is one of the most efficient organic solar cells. In the present study, the structures and electronic states of P3HT-C_60_-PCBM have been investigated by means of the density functional theory (DFT) method to shed light on the mechanism of charge separation in semiconductor materials. The thiophene hexamer was used as a model of P3HT. Five geometrical conformers were obtained as the C_60_-PCBM binary complexes. In the ternary system, P3HT wrapped around C_60_ in the stable structure of P3HT-C_60_-PCBM. The intermolecular distances for P3HT-(C_60_-PCBM) and (P3HT-C_60_)-PCBM were 3.255 and 2.885 Å, respectively. The binding energies of P3HT + (C_60_-PCBM) and (P3HT-C_60_) + PCBM were 27.2 and 19.1 kcal/mol, respectively. The charge transfer bands were found at the low-lying excited states of P3HT-C_60_-PCBM. These bands strongly correlated with the carrier separation and electron transfer in solar cells. The electronic states at the ground and excited states of P3HT-C_60_-PCBM were discussed on the basis of the calculated results.

## 1. Introduction

Solar cells have a low environmental impact, because they convert solar energy directly into electrical energy. Inorganic semiconductor solar cells have reached the realm of practical application. However, the high cost of materials is still an issue. In contrast, solar cells composed of organic thin films are manufactured by applying organic materials to a substrate, which allows for the production of large-area devices at a low cost. Moreover, the flexibility of organic materials makes it possible to manufacture devices that can be bent, depending on the choice of substrate [1,2,3,4,5,6].

The structure of organic thin-film solar cells is similar to that of light-emitting diodes, in which carriers are generally created by bonding p-type and n-type molecules. Recently, devices combining poly(3-hexylthiophene) (P3HT) as the p-type molecule and phenyl C_61_-butyric acid methyl ester (PCBM), a fullerene derivative, as the n-type molecule have been studied [7,8,9,10]. PCBM-P3HT is often used by blending it with C_60_, namely, the P3HT-C_60_-PCBM ternary [11,12,13,14,15].

The heterojunction composed of PCBM-C_60_-P3HT is widely utilized in organic photovoltaic (OPV) solar cells [16,17,18,19]. Richards et al. [20] investigated the doping effects of C_60_ on P3HT-PCBM thin films and revealed that doping C_60_ changed the morphology of P3HT-PCBM. Lee et al. [21] synthesized C_60_-end-capped P3HT (P3HT-C_60_) for the purpose of improving the long-term thermal stability of solar cell performance. When a small amount of P3HT-C_60_ was added to P3HT-PCBM, the bicontinuous and nanometer-scale film morphology was developed and preserved for 2 h of annealing at 150 °C. They showed that the P3HT-PCBM-P3HT-C_60_ bulk heterojunction solar cells exhibited excellent long-term thermal stability in terms of device performance.

From a theoretical approach, the electronic excited states of the complex composed of PCBM and P3HT were investigated by time-dependent density functional theory (TD-DFT) calculations [22]. As a stable structure, the intermolecular distance of PCBM and P3HT was calculated to be 3.5 Å. It was shown that the electronic excitations were composed of polymer internal excitations and charge transfer (CT) bands between the donor and acceptor. Using DFT calculations, Liu and Dennis investigated geometrical conformations of isolated PCBM molecules [23]. A total of 24 possible conformations of PCBM were obtained. Also, their relative energies were determined.

Thus, the electronic states and structures of the P3HT-PCBM system are well understood theoretically. However, the electronic states and interactions of the ternary system composed of P3HT, C_60_, and PCBM, which are important to understand the mechanisms of carrier formation and charge separation in the organic solar cells, are not clearly understood. The electronic states at the excited states of these systems are especially unknown.

In our previous paper [24], we preliminarily investigated the interaction between C_60_ and PCBM using the DFT method in order to elucidate the binding effects of the C_60_PCBM binary complex on solar cells. The binding energy and electronic states were preliminarily estimated. However, the DFT calculations were performed using the PW91 functional without dispersion effects.

In the present study, the ternary system (P3HT-C_60_-PCBM) was investigated by means of the DFT method including dispersion effects in order to obtain the accurate structures and electronic states of the P3HT-C_60_-PCBM system. We focus our attention mainly on the mechanisms of carrier formation and charge separation at the electronic excited states of P3HT-C_60_-PCBM on the basis of accurate structures.

## 2. Method of Calculation

All DFT calculations were performed using the Gaussian 09 program package [25]. First, the geometries of PCBM, C_60,_ and the complexes of C_60_-PCBM were fully optimized at the Austin-Frisch-Petersson functional with dispersion (APFD)/6-31G(d) level. Next, the geometry of the ternary system (Thio-C_60_-PCBM) was optimized, where Thio means the thiophene hexamer and is a model compound of P3HT used in the present calculations. The binding energy of PCBM to C_60_ in binary systems, E_bind_, is defined by the following:

−E_bind_ = E(PCBM-C_60_) − [E(PCBM) + E(C_60_)]

where E(PCBM-C_60_) means the total energy of PCBM-C_60_, and E(PCBM) and E(C_60_) are the total energies of isolated PCBM and C_60_ molecules, respectively. The binding energies, Thio + C_60_-PCBM and Thio-C_60_ + PCBM, were calculated in the ternary system. The atomic and molecular charges of Thio-C_60_-PCBM were obtained by natural population analysis (NPA) at the APFD/6-31G(d) level.

The excitation energies of Thio-C_60_-PCBM were calculated by means of time-dependent (TD) DFT calculations at the CAM-B3LYP/6-31G(d) level. Fifty electronic states were solved. In our previous theoretical calculations, these levels of theory gave reasonable electronic structures of the carbon systems [26,27,28,29,30,31]. In particular, CAM-B3LYP can express the charge transfer (CT) states of molecules.

## 3. Results

### 3.1. (C_60_)_2_ Dimer and (PCBM)_2_ Dimer

**Figure 1:**
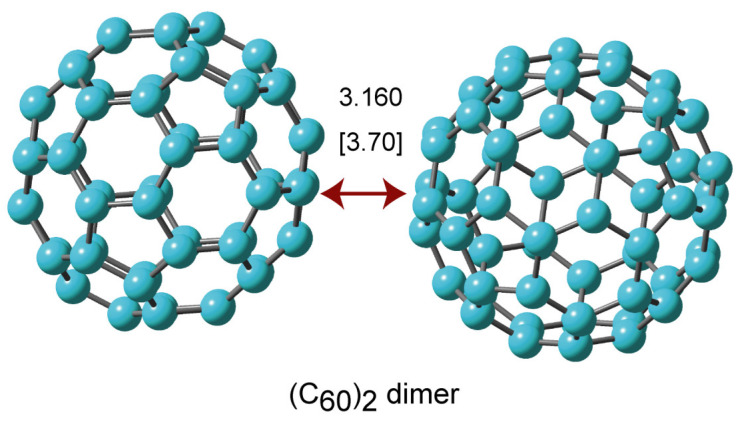
The optimized structure of the (C_60_)_2_ dimer obtained at the APFD/6-31G(d) level. The values are distances in Å.

First, the structure of the C_60_ dimer, expressed as (C_60_)_2_, was fully optimized at the APFD/6-31G(d) level. The calculated structure of (C_60_)_2_ is given in Figure 1. The surfaces of C_60_ molecules interacted with each other (i.e., face-to-face interaction). The value in Figure 1 indicates the intermolecular distance calculated by the APFD method. Also, the value calculated by the PW91 method is given in parenthesis. The intermolecular distances were calculated to be 3.160 Å (APFD) and 3.700 Å (PW91), indicating that the distance in APFD was significantly shorter than that of PW91. The results indicate that dispersion effects (APFD) on the intermolecular distance are important in the C_60_ system. The binding energy of (C_60_)_2_ (i.e., C_60_ + C_60_ → (C_60_)_2_: dimer formation) was calculated to be 13.0 kcal/mol (APDF) and 4.9 kcal/mol (PW91), suggesting that the dispersion effects greatly affected the binding energy between C_60_ molecules. Therefore, hereafter, all calculations were carried out using the APFD functional including dispersion effects.

**Figure 2:**
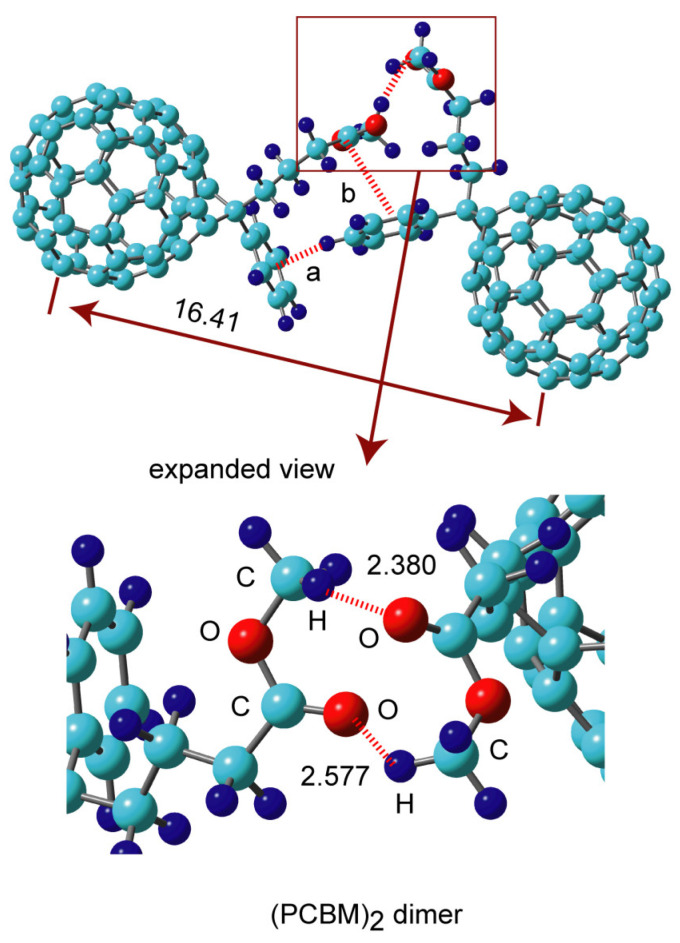
The optimized structure of the (PCBM)_2_ dimer obtained at the APFD/6-31G(d) level. The values are distances in Å.

Similar calculations were carried out for the PCBM dimer, (PCBM)_2_. The optimized structure of (PCBM)_2_ is illustrated in Figure 2. Two PCBM molecules were bound by two hydrogen bonds of the side chain of PCBM, as shown in the expanded view in Figure 2. Carbonyl-proton (C=O–H) hydrogen bonds were formed. The distances of the hydrogen bonds were 2.380 and 2.577 Å. In addition to the two hydrogen bonds, the C-H/π interaction formed bonds between the benzene rings of the two PCBM molecules. This interaction is expressed by “interaction a” in Figure 2. The C-H bond of the benzene ring of one PCBM was oriented toward the π electron of the benzene ring of the neighbor PCBM (interaction a). This interaction is similar to that of a benzene dimer [32,33,34]. Furthermore, interaction between the C=O carbonyl and benzene was also found (interaction b). The binding energy of (PCBM)_2_ was calculated to be 17.1 kcal/mol (APDF), which is larger than that of (C_60_)_2_ (13.0 kcal/mol). This is due to the fact that three interactions exist in (PCBM)_2_ (i.e., hydrogen bond, interaction a and b).

### 3.2. Binary Complexes of C_60_-PCBM

**Figure 3:**
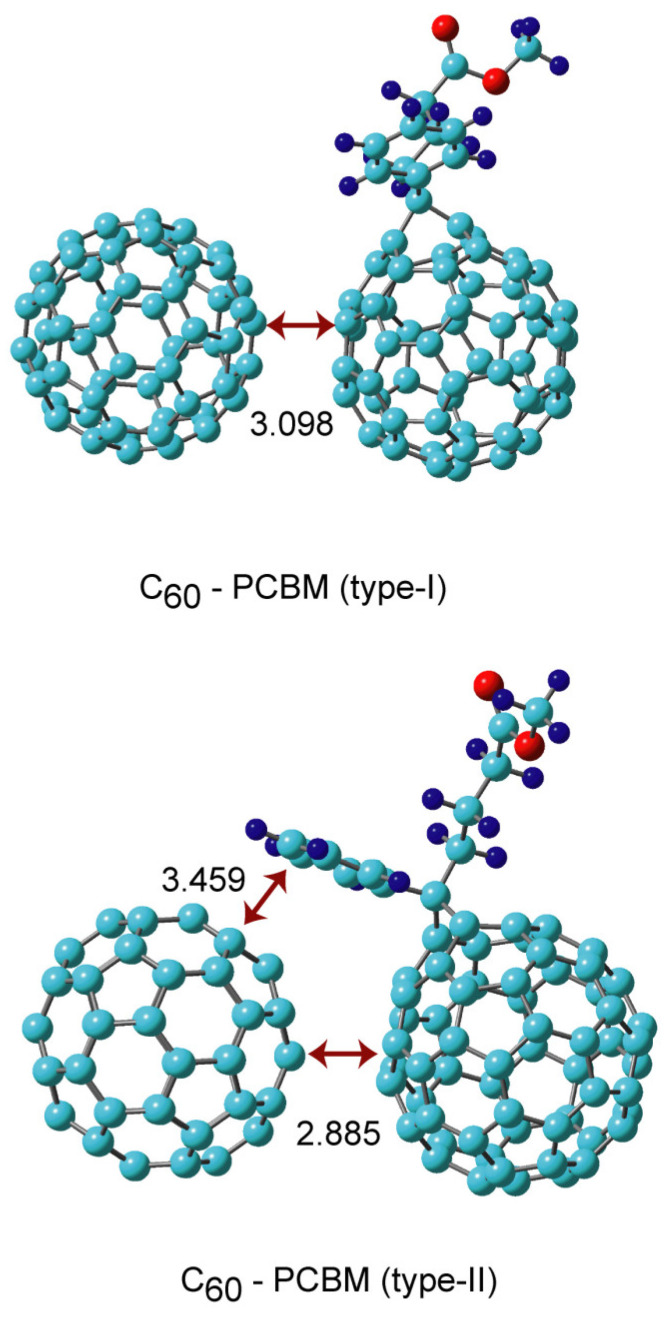
The optimized structures of the C_60_-PCBM complexes (types I and II) obtained at the APFD/6-31G(d) level. The values are distances in Å.

**Figure 4:**
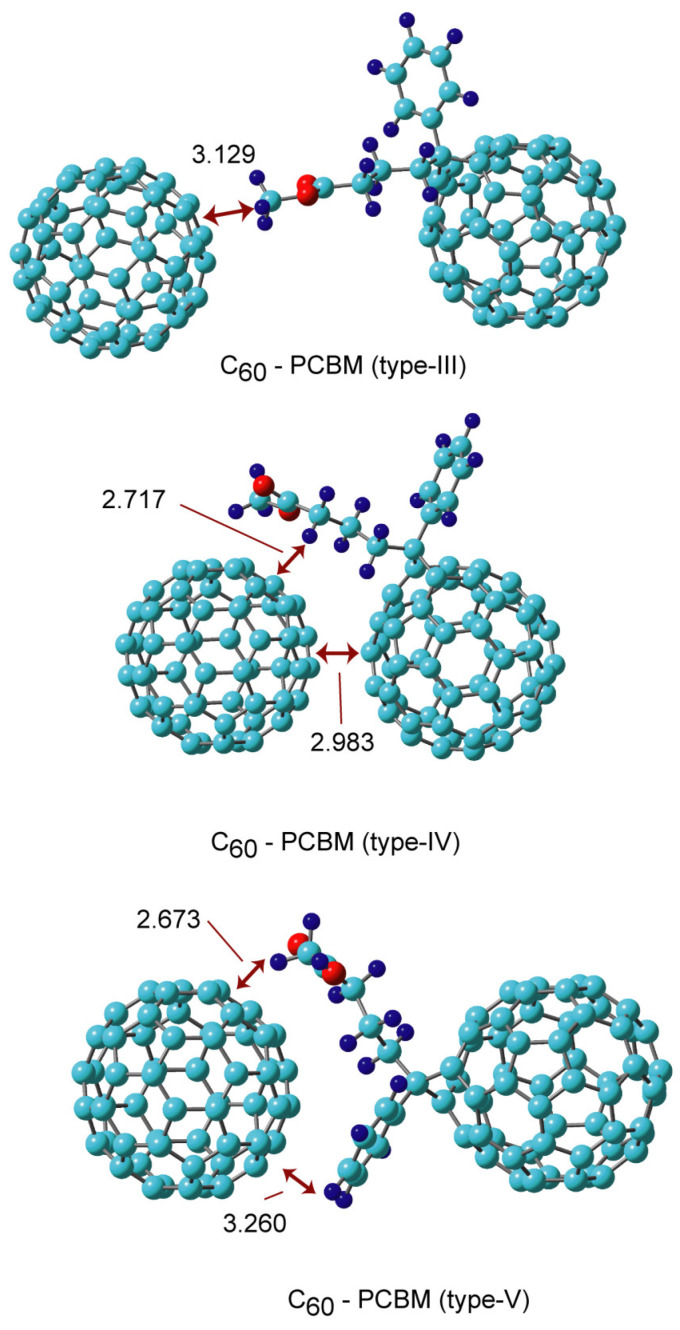
The optimized structures of the C_60_-PCBM complexes (types III, IV, and V) obtained at the APFD/6-31G(d) level. The values are distances in Å.

The geometries of the C_60_-PCBM binary complexes were fully optimized by means of the APFD method based on several initial structures. Five stable structures were obtained, as shown in Figure 3 and Figure 4 (denoted as types I–V). Table 1 shows the calculated binding energies of C_60_-PCBM. In types I and II, the fullerene rings interacted directly with each other in the complexes (face-to-face interaction). The intermolecular distances for types I and II were 3.098 and 2.885 Å, respectively. Only the face-to-face interaction between C_60_ and the C_60_ part of PCBM was found in type I. In contrast, the benzene ring of the side chain of PCBM interacted with C_60_ in type II in addition to the face-to-face interaction.

The binding energies were calculated to be 14.0 kcal/mol (type I) and 19.8 kcal/mol (type II). The former energy was similar to that of the C_60_ dimer (17.1 kcal/mol). The larger binding energy in type II (19.8 kcal/mol) was caused by the π–π stacking interaction between the side-chain benzene of PCBM and C_60_.

In type III, the end of the side chain of PCBM (methyl group) interacted with C_60_. The C-H/π interaction was generated in the complex. The intermolecular distance and binding energy were 3.129 Å and 4.5 kcal/mol, respectively, which was significantly small as a binding energy. This is due to there being no face-to-face interaction in type III.

In type IV, in addition to the face-to-face interaction, the side chains of PCBM were bound to C_60_. In particular, the π-electron in the C=O carbonyl in PCBM interacted with the π-electron of C_60_. The side chain of PCBM flanked C_60_. The binding energy of C_60_ to PCBM was 23.3 kcal/mol. The intermolecular distances were calculated to be 2.717 and 2.983 Å. The binding energy in type IV was the largest of the five complexes. Also, type II had a large binding energy. The intermolecular distances and binding energy were 2.673 and 3.260 Å and 19.6 kcal/mol, respectively, in type V.

The binding energies of all systems are summarized in Table 1, together with those obtained at the APFD/6-311G(d,p)//APFD/6-31G(d) level. Types II and IV showed large binding energies (20.3 and 23.6 kcal/mol, respectively).

### 3.3. Structures and Absorption Spectra of Thiophene-C_60_-PCBM Ternary Complexes

**Figure 5:**
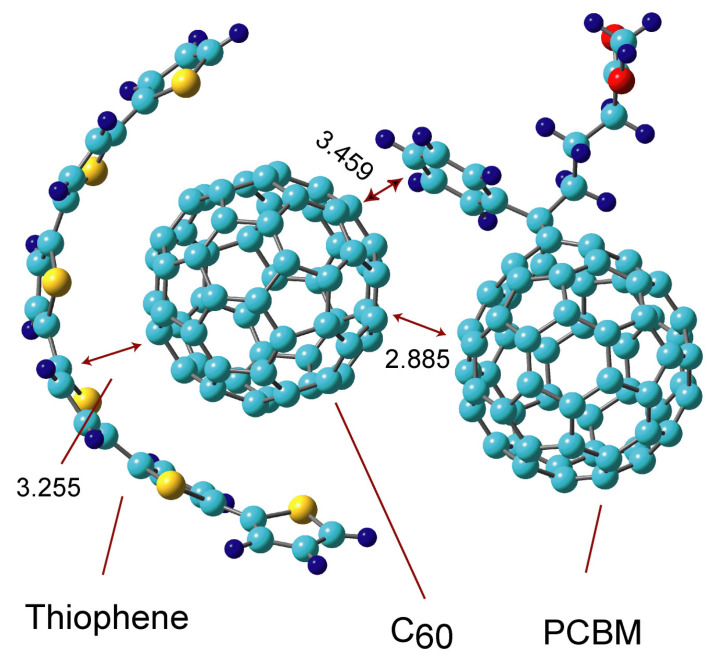
The optimized structures of the thiophene and C_60_-PCBM complex (Thio-C_60_-PCBM) obtained at the APFD/6-31G(d) level. The values are distances in Å.

Next, the structure and electronic states of the ternary system, Thio-C_60_-PCBM, were investigated in this section. The optimized structure for the most stable form is illustrated in Figure 5. The thiophene hexamer (Thio) was employed as a model of P3HT. Thio bound to surround C_60_, where the structure of Thio was deformed largely from the original structure (plane form). The deformation energy of Thio was 14.6 kcal/mol from the plane structure.

The intermolecular distances were 3.255 Å (Thio-C_60_) and 2.885 and 3.459 Å (C_60_-PCBM). The binding energies were 27.2 kcal/mol for Thio + C_60_-PCBM and 19.1 kcal/mol for Thio-C_60_ + PCBM, indicating that the binding of the thiophene oligomer to C_60_ was larger than that of PCBM.

**Figure 6:**
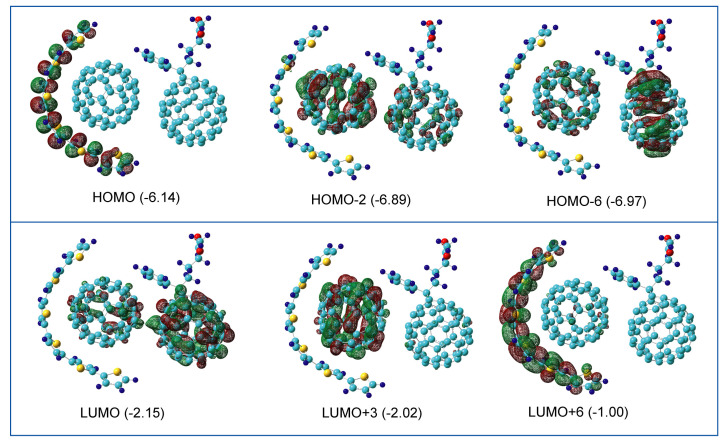
The spatial distributions of the HOMO, LUMO, and MOs around the HOMO and LUMO. The values are orbital energies in eV.

The spatial distributions of molecular orbitals (MOs) of the highest occupied MO (HOMO), the lowest unoccupied MO (LUMO), and the MOs around the HOMO and LUMO are illustrated in Figure 6. The HOMO and the LUMO are localized on Thio and PCBM, respectively. The orbital energies were −6.14 eV (HOMO) and −2.15 eV (LUMO). The MOs of C_60_ were HOMO-2 (occupied: −6.89 eV) and LUMO+3 (unoccupied: −2.02 eV). The MOs of PCBM were HOMO-6 (occupied: −6.97 eV) and LUMO+6 (unoccupied: −1.00 eV).

**Figure 7:**
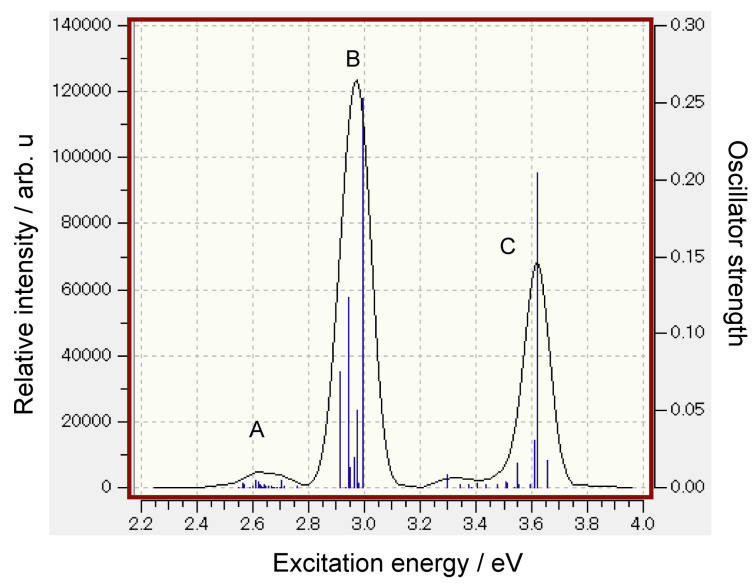
Simulated absorption spectrum of Thio-C_60_-PCBM calculated at CAM-B3LYP/6-31G(d) level.

The simulated absorption spectrum of Thio-C_60_-PCBM is given in Figure 7. The absorption spectra are composed of three bands (A, B, and C). Band A is distributed in the range of 2.45–2.80 eV and is composed of 23 weak electronic excitations. The CTs from Thio to C_60_ and from C_60_ to PCBM were found at a lower energy region (2.56 eV: electron transfer, Thio → C_60_). There was a CT transition from C_60_ to PCBM at 2.59 eV (electron transfer, C_60_ → PCBM). These are neighbor-to-neighbor electron transfers.

Band B is mainly composed of the internal excitation within Thio; the strong peak at 3.00 eV is attributed to a π–π* transition in Thio. Band C is similar to band A, but with high energy excitations. Also, a long-distance CT occurred from Thio to PCBM at the excitation energy of 3.34–3.41 eV. This means that the energy band is widely distributed in the ternary system at the higher energy excited states.

Table 2 shows the ratios of CT transitions in bands A, B, and C. In band A, four CT transitions were found within a total of 23 transitions. The ratio of the CT transition was 0.31 in band A. Band B was mainly composed of internal excitations within molecules (the ratio of CT was 0.09). In band C, the CT transitions increased up to the ratio of 0.38, suggesting that about 40% of electronic transitions are composed of CT transitions.

In the CT states, the charge separations take place, expressed as (thiophene)^+^(C_60_)^−^ and (C_60_)^+^(PCBM)^−^. In the case of higher energy photo-excitation, the long-distance CT occurs also as (Thio)^+^(C_60_)(PCBM)^−^. These results suggest that the charge separation and carrier formation take place easily at low-energy photo-irradiation of the Thio-C_60_-PCBM system. These characteristic features play an important role in the initial stage in solar cells and suggest that Thio-C_60_-PCBM is one of the optimal systems for solar cells.

## 4. Discussion

In the present study, the interaction and absorption spectra in the ternary system, Thio-C_60_-PCBM were investigated by means of the DFT method in order to shed light on the mechanisms of the initial stage in solar cells. Thio-C_60_-PCBM systems are widely used in the core of solar cells.

In the C_60_-PCBM binary complex, the face-to-face interaction and side-chain–C_60_ interaction are important in the formation of the complex. The binding energies are 19.8 kcal/mol (type II) and 23.3 kcal/mol (type IV). In the Thio-C_60_-PCBM ternary system, the structure of thiophene is largely deformed from the original planar structure in the formation of the thiophene-C_60_ complex: C_60_ takes on a structure surrounded by the thiophene. The π-orbital of thiophene is fully overlapped with that of the C_60_’s surface. The nearest intermolecular distances are 3.255 Å for Thio-C_60_ and 2.885 Å for C_60_-PCBM, and the binding energies are 27.2 kcal/mol (Thio-C_60_) and 19.1 kcal/mol (C_60_-PCBM). These values indicate that the binding nature is not composed of pure van der Waals, but also chemical bonds. The molecular orbitals are distributed in the Thio-C_60_-PCBM system. Therefore, the band structure is also distributed in Thio-C_60_-PCBM.

The present calculations show that Thio-C_60_-PCBM has low-energy excitation bands composed of several CT transitions for Thio → C_60_ (2.56 eV) and for C_60_ → PCBM (2.59 eV). In the low-lying excited states of Thio-C_60_-PCBM, CT (namely, electron transfer) takes place, and charge separations occur as (Thio)^+^(C_60_)^−^-PCBM and Thio-(C_60_)^+^(PCBM)^−^. These charge separations cause the formation of carriers in solar cells. In the Thio-C_60_-PCBM system, this process takes place easily at low-energy photo-irradiation.

Similar CT transitions were previously measured as absorption spectra of the complexes of C_60_-(aromatic amines). Ichida et al. observed that the excitation energies were 2.19 eV (C_60_-*N*,*N*-diethylaniline), 2.33 eV (C_60_-*N*,*N*-dimethylaniline), and 2.48 eV (C_60_-*N*-methylaniline) [35,36]. These excitation energies of the CT transitions were in agreement with the excitation energies in the Thio-C_60_-PCBM system (2.5–2.8 eV in band A). These agreements support the validity of the present calculations. Thus, the present study has provided important insights into the mechanisms of the initial stage of solar cells.

**Figure 8:**
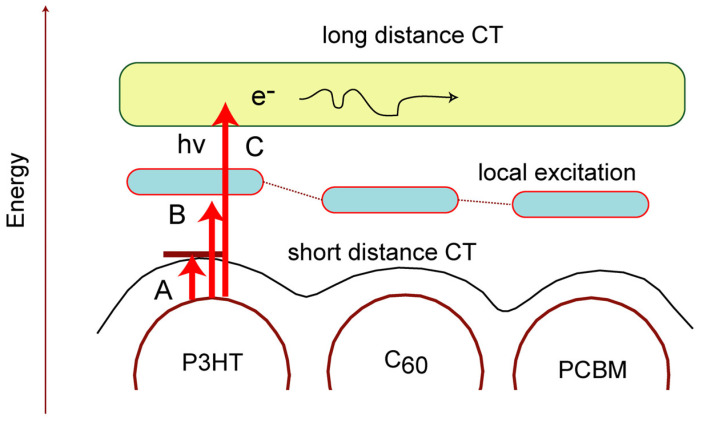
Schematic illustration of band structure of Thio-C_60_-PCBM and assignments of excitation bands.

As a summary of the TD calculations, a schematic illustration of the band structure of Thio-C_60_-PCBM is given. Figure 8 shows the band structure of Thio-C_60_-PCBM and the assignments of electronic excitations. The low-energy excitation (band A in Figure 8) causes an electron transfer from Thio to C_60_ or from C_60_ to PCBM (i.e., a short-distance electron transfer). The middle-energy excitation (band B) causes a local internal excitation within each molecule. The high-energy excitation (band C) causes a long-distance electron transfer. In the Thio-C_60_-PCBM system, the charge separation takes place easily after the photo-irradiation.

Conversion efficiency is extremely important for photovoltaics. The most popular Si cells achieve an efficiency of 26.7% (mono-crystalline laboratory set) and 24.4% (multi-crystalline Si wafers) [37]. The other material cells, using thin-film technology, offer growing efficiencies up to 23.4% for copper indium gallium selenide (CIGS) and 21.0% for cadmium telluride (CdTe) solar cells. A record efficiency of 25.6% was obtained for a single-junction perovskite cell in 2021. The organic thin-film solar cells have a maximum photoelectric energy conversion efficiency of about 8–10% [38], which is less than the conversion efficiencies of amorphous, polycrystalline, and monocrystalline silicon solar cells.

For future research directions, we propose the following. There are many molecules similar to PCBM, such as C_70_-PCBM. The electronic structures of the similar molecules could be clarified by DFT calculations and the best molecule could be predicted from theoretical points of view. In the present study, we also showed that the wrapped structure of P3HT around the fullerene surface is important in the interaction of P3HT-C_60_. Halogen substitution (for example, F atom) in P3HT may change the binding energy and winding structure of P3HT-C_60_. The above directions would have the potential to improve the performance of solar cells.

## 5. Conclusions

In order to develop new solar cells, it is important to understand the mechanisms of existing solar cells. The ternary system, composed of poly(3-hexylthiophene) (P3HT), fullerene (C_60_), and phenyl-C_61_-butyric-acid-methyl-ester (PCBM), expressed as P3HT-C_60_-PCBM, is one of the most efficient organic solar cells. In the present study, the structures and electronic states of P3HT-C_60_-PCBM have been investigated by means of the DFT method to shed light on the mechanisms of solar cells composed of semiconductor materials. First, the structures of the binary complexes of C_60_-PCBM were calculated from several initial conformations. Five stable structures were obtained (types I–V). Next, the complexes of the thiophene oligomer (Thio) with C_60_-PCBM were calculated.

Using the most stable complex of Thio-C_60_-PCBM, the TD-DFT calculations were carried out. CT bands were found at the low-lying excited states of P3HT-C_60_-PCBM. These CT bands showed that charge separations, (P3HT)^+^-(C_60_)^−^-PCBM, P3HT-(C_60_)^+^-(PCBM)^−^, and (P3HT)^+^-C_60_-(PCBM)^−^, occurred due to photo-irradiation. In the lowest energy band (band A in Figure 8), a neighbor-to-neighbor CT takes place (i.e., a short-distance electron transfer). The middle-energy band (B) is composed of the internal local excitations within the molecules. In the higher energy band (C), a long-distance electron transfer occurs. The energy bands strongly correlate with the carrier generation and electron transfer in solar cells.

## Figures and Tables

**Table 1:** The binding energies (E_b_ in kcal/mol) of the (C_60_)_2_ dimer, (PCBM)_2_ dimer, and C_60_-PCBM complexes (types I–V) calculated at the APDF/6-31G(d) level. The binding energies obtained at the APFD/6-311G(d,p)//APFD/6-31G(d) are also given as E_b_(6-311).

System	E_b_/kcal mol^−1^	E_b_(6-311)/kcal mol^−1^
(PCBM)_2__dimer	13.04	13.37
(C_60_)_2__dimer	17.13	16.71
C_60_-PCBM (I)	14.02	14.44
C_60_-PCBM (II)	19.81	20.31
C_60_-PCBM (III)	4.51	4.46
C_60_-PCBM (IV)	23.28	23.64
C_60_-PCBM (V)	19.57	19.67

**Table 2:** Ratios of CT transitions in bands A, B, and C. Numbers of CT, internal, and total transitions in electronic excitations.

Band	Energy Region/eV	CT	Internal	Total	Ratio of CT
A	2.5–2.8	4	19	23	0.31
B	2.9–3.1	1	10	11	0.09
C	3.3–3.8	6	10	16	0.38

## Data Availability

Data are contained within the article.

## References

[1] Chen R., Yan Y., Wang X., Chang C., Zhao Y., Liu Y., Wei D.J. (2022). Patterning an Erosion-Free Polymeric Semiconductor Channel for Reliable All-Photolithography Organic Electronics. J. Phys. Chem. Lett..

[2] Chetyrkina M.R., Talalaev F.S., Kameneva L.V., Kostyukb S.V., Troshin P.A. (2022). Vat dyes: Promising biocompatible organic semiconductors for wearable electronics applications. J. Mater. Chem. C.

[3] Lin Y., Fan H., Li Y., Zhan X. (2012). Organic Semiconductors: Thiazole-Based Organic Semiconductors for Organic Electronics. Adv. Mater..

[4] Clancy P. (2012). Chemical engineering in the electronics industry: Progress towards the rational design of organic semiconductor heterojunctions. Curr. Opin. Chem. Eng..

[5] Hlawacek G., Khokhar F.S., van Gastel R., Poelsema B., Teichert C. (2011). Smooth Growth of Organic Semiconductor Films on Graphene for High-Efficiency Electronics. Nano Lett..

[6] Xie L.-H., Yin C.-R., Lai W.-Y., Fan Q.-L., Huang W. (2012). Pd- and Ni-catalyzed cross-coupling reactions in the synthesis of organic electronic materials. Prog. Polym. Sci..

[7] Khairulaman F.L., Yap C.C., Jumali M.H.H., Issa N.A. (2022). Pengoptimuman Lapisan P3HT:PCBM Terdop CuI dalam Sel Suria Organik Jenis Songsang untuk Aplikasi Cahaya Dalam. Sains Malays..

[8] Boudreault P.-L.T., Hennek J.W., Ortiz R.P., Eckstein B.J., Facchetti A., Marks T.J. (2012). New Semiconductors Based on 2,2′-Ethyne-1,2-diylbis[3-(alk-1-yn-1-yl)thiophene] for Organic Opto-Electronics. Chem. Mater..

[9] Sahoo S., Barah D., Kumar S D., Xavier N., Dutta S., Ray D., Bhattacharyya J. (2022). The nature of excitons in PPDT2FBT:PCBM solar cells: Role played by PCBM. Phys. D Appl. Phys..

[10] Kalkan Y., Öztürk S., Kösemen A. (2022). Effects of PCBM loading on high sensitive P3HT based vertical bulk resistive X-ray detector. Org. Electron..

[11] Gami F., Guizani I., Sebak M.A., Abuelwafa A.A., Mostafa M.M. (2022). Investigation of structural, optical and electrical properties of PCBM/ZnOEP thin films. Opt. Mater..

[12] Munshi J., Chen W., Chien T.-Y., Balasubramanian G. (2021). Machine learned metaheuristic optimization of the bulk heterojunction morphology in P3HT:PCBM thin films. Comput. Mater. Sci..

[13] Singh V., Kumar R. (2019). Fowler Nordheim Plot Analysis of Degradation in P3HT:PCBM Thin Film MIM Devices. Macromol. Res..

[14] Huq A.F., Karim A. (2019). Comparative solvent quality dependent crystallization in solvent vapor annealing of P3HT:PCBM thin films by in-situ GIWAXS. Polymer.

[15] Pont S., Foglia F., Higgins A.M., Durrant J.R., Cabral J.T. (2018). Stability of Polymer:PCBM Thin Films under Competitive Illumination and Thermal Stress. Adv. Funct. Mater..

[16] Hibner-Kulicka P., Waliszewski W., Borkowski M., Luszczynska B., Szymanski M., Marszalek T., Ulanski J. (2019). Influence of P3HT preaggregation process on performance of the P3HT:C_60_-PCBM solar cells. Mol. Cryst. Liq. Cryst..

[17] Xu Y., Huang X., Yuan J., Ma W. (2018). From PCBM-Polymer to Low-Cost and Thermally Stable C_60_/C_70_-Polymer Solar Cells: The Role of Molecular Structure, Crystallinity, and Morphology Control Click to copy article link. ACS Appl. Mater. Interfaces.

[18] Namkoong G., Mamun A.A., Ava T.T. (2018). Impact of PCBM/C_60_ electron transfer layer on charge transports on ordered and disordered perovskite phases and hysteresis-free perovskite solar cells. Org. Electron..

[19] Nardes A.M., Ferguson A.J., Whitaker J.B., Larson B.W., Larsen R.E., Maturová K., Graf P.A., Boltalina O.V., Strauss S.H., Kopidakis N. (2012). Beyond PCBM: Understanding the Photovoltaic Performance of Blends of Indene-C_60_ Multiadducts with Poly(3-hexylthiophene). Adv. Funct. Mater..

[20] Richards J.J., Rice A.H., Nelson R.D., Kim F.S., Jenekhe S.A., Luscombe C.K., Pozzo D.C. (2013). Modification of PCBM crystallization via incorporation of C_60_ in polymer/fullerene solar cells. Adv. Funct. Mater..

[21] Lee J.U., Jung J.W., Emrick T., Russell T.P., Jo W.H. (2010). Synthesis of C_60_-end capped P3HT and its application for high performance of P3HT/PCBM bulk heterojunction solar cells. J. Mater. Chem..

[22] Van den Brande N., Patil N., Guizar-Sicairos M., Claessens R., Van Assche G., Breiby D.W., Van Mele B. (2017). Probing the bulk heterojunction morphology in thermally annealed active layers for polymer solar cells. Org. Electron..

[23] Liu T., Dennis T.J.S. (2021). Conformational Analysis of [60]PCBM from DFT Simulations of Electronic Energies, Bond Strain and the ^13^C NMR Spectrum: Input Geometry Determination and Ester Bond Rotation Dynamics. C.

[24] Abe S., Tachikawa H., Iyama T., Safaee S., Nesabi M., Valanezhad A., Watanabe I. (2024). Density functional theory study on the interaction of C_60_ fullerene with PCBM. Jpn. J. Appl. Phys..

[25] Frisch M.J., Trucks G.W., Schlegel H.B., Scuseria G.E., Robb M.A., Cheeseman J.R., Montgomery J.A., Vreven T., Kudin K.N., Burant J.C. (2003). Ab-Initio Calculation Program: Gaussian 09, Revision B.04.

[26] Tachikawa H., Iyama T. (2019). Mechanism of Hydrogen Storage in the Graphene Nanoflake–Lithium–H_2_ System. J. Phys. Chem. C.

[27] Tachikawa H., Yi H., Iyama T., Yamasaki S., Azumi K. (2022). Hydrogen Storage Mechanism in Sodium-Based Graphene Nanoflakes: A Density Functional Theory Study. Hydrogen.

[28] Tachikawa H., Iyama T. (2022). Reactions of Graphene Nano-Flakes in Materials Chemistry and Astrophysics. PhysChem.

[29] Tachikawa H. (2017). Hydrogen atom addition to the surface of graphene nanoflakes: A density functional theory study. Appl. Surf. Sci..

[30] Tachikawa H. (2016). Ionization dynamics of water dimer on ice surface. Surf. Sci..

[31] Tachikawa H. (2022). Mechanism of Li storage on graphene nanoflakes: Density functional theory study. Surf. Sci..

[32] Tachikawa H., Iura R., Kawabata H. (2019). Water-accelerated π-Stacking Reaction in Benzene Cluster Cation. Sci. Rep..

[33] Tachikawa H. (2018). Jahn–Teller Effect of the Benzene Radical Cation: A Direct ab Initio Molecular Dynamics Study. J. Phys. Chem. A.

[34] Tachikawa H., Miyazawa Y., Iura R. (2018). Timescale of π-Stacking Formation in a Benzene Trimer Cation Formed by Ionization of the Parent Neutral Trimer: A Direct Ab Initio Molecular Dynamics Study. Chem. Select..

[35] Ichida M., Sohda T., Nakamura A. (2000). Third-Order Nonlinear Optical Properties of C_60_ CT Complexes with Aromatic Amines. J. Phys. Chem. B.

[36] Ichida M., Sohda T., Nakamura A. (1999). Optical Transition and Ionicity of C_60_/Amine Charge-Transfer Complexes Studied by Optical Spectroscopy. Chem. Phys. Lett..

[37] Jacak J.E., Jacak W.A. (2022). Routes for metallization of perovskite solar cells. Materials.

[38] Vohra V., Kawashima K., Kakara T., Koganezawa T., Osaka I., Takimiya K., Murata H. (2015). Efficient inverted polymer solar cells employing favourable molecular orientation. Nat. Photonics.

